# Synthesis, Antiplatelet Activity and Cytotoxicity Assessment of Indole-Based Hydrazone Derivatives

**Published:** 2015

**Authors:** Kamaleddin Haj Mohammad Ebrahim Tehrani, Marjan Esfahani Zadeh, Vida Mashayekhi, Maryam Hashemi, Farzad Kobarfard, Farhad Gharebaghi, Shohreh Mohebbi

**Affiliations:** a ***Zanjan Pharmaceutical Biotechnology Research Center, ****Zanjan University of Medical Sciences, Zanjan, Iran****.***; b *Department of Medicinal Chemistry, School of Pharmacy, Shahid Beheshti University of Medical Sciences, Tehran, Iran. *; c *Department of Pharmaceutical Biotechnology, School of Pharmacy, Zanjan University of Medical Sciences, Zanjan, Iran. *; d *Department of Medicinal Chemistry, School of Pharmacy, Zanjan University of Medical Sciences, Zanjan, Iran.*

**Keywords:** Indole, Arylhydrazone, Antiplatelet aggregation, Arachidonic acid, Collagen

## Abstract

A series of indole-based aryl(aroyl)hydrazone analogs of antiplatelet indole-3-carboxaldehyde phenylhydrazone were synthesized by the Schiff base formation reaction and their antiplatelet activity was assessed using human platelet rich plasma. The platelet concentrate was obtained using a two-step centrifugation protocol and ADP, arachidonic acid and collagen were used as inducers of platelet aggregation. Based on the results, substituted phenylhydrazones showed promising activity. Among them, compound 1i was the most potent derivative with an IC_50_ comparable to that of indomethacin as a standard drug. The hydrazone derivatives were also tested for their cytotoxicity using on platelet concentrates and fibroblast L929 cells. The majority of the derivatives showed an acceptable selectivity towards antiplatelet aggregation activity.

Based on the activity data, phenylhydrazone derivatives (1a-i) exhibited considerable antiplatelet activity and minimal toxic effect on platelet cells. The results of the present study could provide a better understanding of the structure activity relationship of antiplatelet indolehydrazones.

## Introduction

As a major cause of death and disability, thromboembolic disorders are a serious health problem in worldwide and even in industrial countries(1). It has been revealed that increased self-affinity and aggregation of platelets play animportant role in the pathogenesis of atherothrombosis(2). To prevent such a condition, antiplatelet agents including aspirin and clopidogrelare employed in the drug therapy regimens. However, there are serious limitations to these agents including increased risk of bleeding(3) and drug resistance(4). Therefore, discovery and development of new antiplatelet agents with improved efficacy and safety are an urgent need.

In the published works focused on the development of novel antiplatelet agents, a variety of hydrazone derivatives have been reported. The antiplatelet aryl(aroyl)hydrazones of aromatic aldehydes and methyl ketones reported by Coquelet et al.(5) and diverse groups of hydrazones including arylsulfonateacylhydrazones(6), phenothiazine-1-acylhydrazones(7)*N*-substituted-phenyl-1,2,3-triazole-4-acylhydrazones(8) and pyrazolylhydrazones(9) are some examples of hydrazone derivatives with potent antiplatelet aggregation activities.

**Figure 1:**
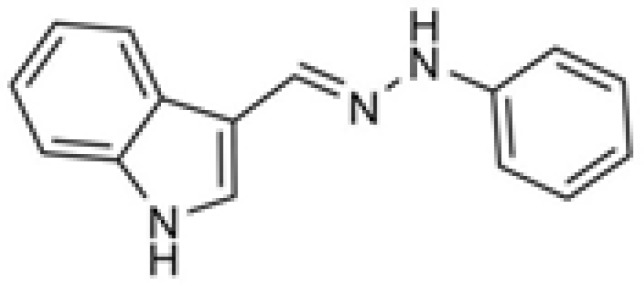
The antiplatelet indole-3-carboxaldehyde phenylhydrazone

Based on the above mentioned reports, we have investigated the antiplatelet activity of some novel indole and hydrazone derivatives(10,11, 12). Focused on the bioscreening of hydrazone derivatives of aromatic aldehydes, we found some arylhydrazones of electron rich heteroaromatic aldehydes as the most potent derivatives among the studied compounds. Of the most potent derivatives, indole-3-carboxaldehyde phenylhydrazone exhibited excellent inhibition of platelet aggregation induced by arachidonic acid (IC_50_ = 10 µM, Figure 1).This compound has a relatively small molecular weight (235.28) and an optimal choice to be considered as lead compound.

**Figure 2:**
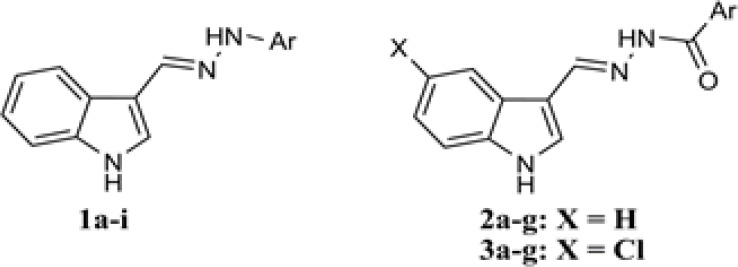
General structure of the synthesized derivatives

Therefore, in the present study,in order to better understand the structure-activity relationship of indole-based hydrazones and also to discover new derivatives with improved antiplatelet activity, we synthesized some new hydrazone derivatives of indole-3-carboxaldehyde and assessed their inhibitory activity against arachidonic acid-, ADP- and collagen-induced platelet aggregation. As shown in figure 2, the derivatives have been divided in 3 subgroups including arylhydrazones of indole-3-carboxaldehyde (1a-i), aroylhydrazones of indole-3-carboxaldehyde (2a-g) and aroylhydrazones of 5-chloroindole-3-carboxaldehyde (3a-g). Different substitutions have been made on the aromatic ring to evaluate the relationship between different physicochemical parameters and the observed bioactivities.

In addition to antiplatelet activity, the synthesized derivatives were evaluated for their potential toxicity on a normal cell line (L929) and on platelet itself (platelet membrane leakage assay).

## Experimental

*Materials and Methods*

Melting points were measured by an Electrothermal 9200 apparatus and were uncorrected. The ^1^H-NMR spectra were measured by a 400 MHz BrukerAvance DRX spectrometer in *d*_6_-DMSO and TMS was used as an internal standard. The infrared spectra were obtained by a Bruker Tensor27ATR-FTIR spectrophotometer and samples were directly used without the addition of KBr. ESI-MS spectra were obtained by Agilent 6410 Triple Quad LC/MS. The derivatives were analyzed for C, H, N by a Costech model 4010 and the obtained values were in agreement with the proposed structures within ±0.4% of the theoretical values.The synthesis and spectral characterization of compounds 1a(11), 1b, 1e, 1f(13), 3a(14)and 3c(15) are available in the literature.Calculation of the global physicochemical parameters including ClogP, polarizability (P), refractivity (R), surface area (SA) and molecular volume (V) were performed using Hyperchem 8.0 software.

*General procedure for the synthesis of hydrazone derivatives*

(5-Chloro)Indole-3-carboxaldehyde and appropriate arylhydrazines/aroylhydrazides (1 mmol of each) were added to a mixture of ethanol (96%, 10 mL) and glacial acetic acid (8 drops). The resulting mixture was heated under reflux and the reaction progress was monitored by thin layer chromatography. The mixture was then cooled to room temperature. The precipitate was filtered, washed with *n*-hexane and dried in the open air to afford the final derivatives.

*Indole-3-carboxaldehyde 2-chlorophenylhydrazone (1c)*

Yield 69%,m.p. 150-153°C.^1^HNMR (400 MHz, DMSO-*d*_6_) *δ*: 6.72 (dt, *J* = 8.0 Hz, *J* = 1.6 Hz, 1H, Ar H), 7.14-7.21 (m, 2H, indole C_5,6_-H), 7.29 (m, 2H, Ar H), 7.42 (dd, *J* = 6.8 Hz, *J* = 2.0 Hz, 1H, indole C_7_-H), 7.54 (d, *J* = 7.6 Hz, 1H, Ar H), 7.65 (d, *J* = 2.8 Hz, 1H, indole C_2_-H), 8.23 (d, *J* = 7.6 Hz, 1H, indole C_4_-H), 8.49 (s, 1H, imine H), 9.37 and 11.41 (each s, 1H, NH); IR (cm^-1^): 3344, 1597, 1438, 1240, 736. ESI-MS *m/z*: 270, 272 (M + H^+^). Anal.Calcd for C_15_H_12_ClN_3_: C, 66.79; H, 4.48; N, 15.58. Found: C, 66.67; H, 4.47; N, 15.60.

*Indole-3-carboxaldehyde 3-chlorophenylhydrazone (1d)*

Yield 77%,m.p. 211-214°C.^1^HNMR (400 MHz, DMSO-*d*_6_)*δ*: 6.67 (d, *J* = 8.00 Hz, 1H, Ar C_6_-H), 6.94 (d, *J* = 8.4 Hz, 1H, Ar C_4_-H), 7.01 (s, 1H, Ar C_2_-H), 7.14-7.23 (m, 3H, indole C_5,6_-H + Ar C_5_-H), 7.42 (d, *J* = 7.6 Hz, 1H, indole C_7_-H), 7.66 (d, *J* = 2.4 Hz, 1H, indole C_2_-H), 8.12 (s, 1H, imine H), 8.18 (d, *J* = 7.6 Hz, 1H, indole C_4_-H), 10.04 and 11.37 (each s, 1H, NH); IR (cm^-1^): 3415, 1589, 1530, 1426, 1083, 750, 682. ESI-MS *m/z*: 270, 272 (M + H^+^). Anal.Calcd for C_15_H_12_ClN_3_: C, 66.79; H, 4.48; N, 15.58. Found: C, 66.68; H, 4.48; N, 15.57.

*Indole-3-carboxaldehyde 2-methylphenylhydrazone (1g)*

Yield 83%,m.p. 176-178°C.^1^HNMR (400 MHz, DMSO-*d*_6_)*δ*: 2.20 (s, 3H, CH_3_), 6.64 (t, *J* = 7.2 Hz, 1H, Ar H), 7.02 (d, *J* = 7.2 Hz, 1H, Ar H), 7.12-7.19 (m, 3H, indole C_5,6_-H + Ar H), 7.42 (m, 2H, indole C_7_-H + Ar H), 7.62 (d, *J* = 2.4 Hz, 1H, indole C_2_-H), 8.24 (d, *J* = 6.8 Hz, 1H, indole C_4_-H), 8.37 (s, 1H, imine H), 8.99 and 11.33 (each s, 1H, NH); IR (cm^-1^): 3242, 1601, 1531, 1242, 1118, 1074. ESI-MS *m/z*: 250 (M + H^+^). Anal.Calcd for C_16_H_15_N_3:_ C, 77.08; H, 6.06; N, 16.85. Found: C, 77.17; H, 6.05; N, 16.87.

*Indole-3-carboxaldehyde 4-methylphenylhydrazone (1h)*

Yield 89%,m.p. 228-231°C.^1^HNMR (400 MHz, DMSO-*d*_6_)*δ*: 2.20 (s, 3H, CH_3_), 6.93 (d, *J* = 8.2 Hz, 2H, Ar C_3,5_-H), 7.02 (d, *J* = 8.2 Hz, 2H, Ar C_2,6_-H), 7.11-7.18 (m, 2H, indole C_5,6_-H), 7.39 (d, *J* = 7.2 Hz, 1H, indole C_7_-H), 7.59 (d, *J* = 2.4 Hz, 1H, indole C_2_-H), 8.07 (s, 1H, imine H), 8.22 (d, *J* = 7.2 Hz, 1H, indole C_4_-H), 9.65 and 11.27 (each s, 1H, NH); IR (cm^-1^): 3296, 1608, 1242, 1079, 809, 751. ESI-MS *m/z*: 250 (M + H^+^). Anal.Calcd for C_16_H_15_N_3_: C, 77.08; H, 6.06; N, 16.85. Found: C, 77.15; H, 6.06; N, 16.82.

*Indole-3-carboxaldehyde 4-methoxyphenylhydrazone (1i)*

Yield 75%,m.p. 200-203°C.^1^HNMR (400 MHz, DMSO-*d*_6_)*δ:*3.68 (s, 3H, OCH_3_), 6.86 (d, *J* = 8.4 Hz, 2H, Ar C_3,5_-H), 6.96 (d, *J* = 8.4 Hz, 2H, Ar C_2,6_-H), 7.10-7.18 (m, 2H, indole C_5,6_-H), 7.39 (d, *J* = 6.8 Hz, 1H, indole C_7_-H), 7.57 (d, *J* = 2.8 Hz, 1H, indole C_2_-H), 8.06 (s, 1H, imine H), 8.23 (d, *J* = 7.2 Hz, 1H, indole C_4_-H), 9.53 and 11.26 (each s, 1H, NH); IR (cm^-1^): 3285, 1610, 1511, 1441, 1240, 822. ESI-MS *m/z*: 266 (M + H^+^). Anal.Calcd for C_16_H_15_N_3_O: C, 72.43; H, 5.70; N, 15.84. Found: C, 72.31; H, 5.68; N, 15.89.

*Indole-3-carboxaldehyde benzoylhydrazone (2a)*

Yield 90%,m.p. 238-241°C.^1^HNMR (400 MHz, DMSO-*d*_6_)*δ*: 7.13-7.22 (m, 2H, indole C_5,6_-H), 7.43 (d, *J* = 7.6 Hz, 1H, indole C_7_-H), 7.49-7.58 (m, 3H, Ar C_3,4,5_-H), 7.81 (s, 1H, indole C_2_-H), 7.91 (d, *J* = 7.2 Hz, 2H, Ar C_2,6_-H), 8.29 (d, *J* = 7.6 Hz, 1H, indole C_4_-H), 8.61 (s, 1H, imine H), 11.49 and 11.56 (each s, 1H, NH); IR (cm^-1^): 3209, 1603, 1575, 1443, 720. ESI-MS *m/z*: 264 (M + H^+^). Anal.Calcd for C_16_H_13_N_3_O: C, 72.99; H, 4.98; N, 15.96. Found: C, 73.06; H, 4.98; N, 15.92.

*Indole-3-carboxaldehyde 3-chlorobenzoylhydrazone (2b)*

Yield 78%,m.p. 215-218°C.^1^HNMR (400 MHz, DMSO-*d*_6_)*δ:*7.13-7.22 (m, 2H, indole C_5,6_-H), 7.43 (d, *J* = 8.0 Hz, 1H, indole C_7_-H), 7.56 (t, *J* = 8.0 Hz, 1H, Ar C_5_-H), 7.64 (d, *J* = 8.4 Hz, 1H, Ar H), 7.83 (s, 1H, indole C_2_-H), 7.88 (d, *J* = 8.4 Hz, 1H, Ar H), 7.96 (s, 1H, Ar C_2_-H), 8.28 (d, *J* = 7.6 Hz, 1H, indole C_4_-H), 8.60 (s, 1H, imine H), 11.52 and 11.59 (each s, 1H, NH); IR (cm^-1^): 3259, 1603, 1357, 879, 680. ESI-MS *m/z:* 298, 300 (M + H^+^). Anal.Calcd for C_16_H_12_ClN_3_O: C, 64.54; H, 4.06; N, 14.11. Found: C, 64.45; H, 4.07; N, 14.13.

*Indole-3-carboxaldehyde 4-chlorobenzoylhydrazone (2c)*

Yield 86%,m.p. 230-233°C^1^HNMR (400 MHz, DMSO-*d*_6_)*δ:*7.12-7.21 (m, 2H, indole C_5,6_-H), 7.43 (d, *J* = 8.0 Hz, 1H, indole C_7_-H), 7.59 (d, *J* = 8.4 Hz, 2H, Ar H), 7.82 (s, 1H, indole C_2_-H), 7.94 (d, *J* = 8.4 Hz, 2H, Ar H), 8.28 (d, *J* = 8.0 Hz, 1H, indole C_4_-H), 8.60 (s, 1H, imine H), 11.56 (s, 2H, NH); IR (cm^-1^): 3341, 1617, 1595, 1354, 1097, 843. ESI-MS *m/z*: 298, 300 (M + H^+^). Anal.Calcd for C_16_H_12_ClN_3_O: C, 64.54; H, 4.06; N, 14.11. Found: C, 64.40; H, 4.07; N, 14.16.

*Indole-3-carboxaldehyde 4-nitrobenzoylhydrazone (2d)*

Yield 71%,m.p. 250-253°C.^1^HNMR (400 MHz, DMSO-*d*_6_)*δ:*7.14-7.23 (m, 2H, indole C_5,6_-H), 7.44 (d, *J* = 7.6 Hz, 1H, indole C_7_-H), 7.86 (s, 1H, indole C_2_-H), 8.15 (d, *J* = 8.8 Hz, 2H, Ar C_3,5_-H), 8.29 (d, *J* = 7.6 Hz, 1H, indole C_4_-H), 8.36 (d, *J* = 8.8 Hz, 2H, Ar C_2,6_-H), 8.63 (s, 1H, imine H), 11.62 and 11.79 (each s, 1H, NH); IR (cm^-1^): 3336, 1639, 15595, 1350, 744. ESI-MS *m/z*: 309 (M + H^+^). Anal.Calcd for C_16_H_12_N_4_O_3_: C, 62.33; H, 3.92; N, 18.17. Found: C, 63.41; H, 3.92; N, 18.11.

*Indole-3-carboxaldehyde 3-methylbenzoylhydrazone (2e)*

Yield 74%,m.p. 212-214°C.^1^HNMR (400 MHz, DMSO-*d*_6_)*δ*: 2.39 (s, 3H, CH_3_), 7.14-7.20 (m, 2H, indole C_5,6_-H), 7.38-7.44 (m, 3H, Ar C_4,5_-H + indole C_7_-H), 7.70 (d, *J* = 6.8 Hz, 1H, Ar C_6_-H), 7.72 (s, 1H, Ar C_2_-H), 7.80 (s, 1H, indole C_2_-H), 8.29 (d, *J* = 7.6 Hz, 1H, indole C_4_-H), 8.60 (s, 1H, imine H), 11.44 and 11.56 (each s, 1H, NH); IR (cm^-1^): 3205, 1600, 1575, 708. ESI-MS *m/z*: 278 (M + H^+^). Anal.Calcd for C_17_H_15_N_3_O: C, 73.63; H, 5.45; N, 15.15. Found: C, 73.52; H, 5.44; N, 15.14.

*Indole-3-carboxaldehyde 4-methylbenzoylhydrazone (2f)*

Yield 88%,m.p. > 270°C.^1^HNMR (400 MHz, DMSO-*d*_6_)*δ:*2.37 (s, 3H, CH_3_), 7.12-7.21 (m, 2H, indole C_5,6_-H), 7.31 (d, *J* = 8.0 Hz, 2H, Ar C_2,6_-H), 7.43 (d, *J* = 8.0 Hz, 1H, indole C_7_-H), 7.79 (s, 1H, indole C_2_-H), 7.83 (d, *J* = 8.0 Hz, 2H, Ar C_3,5_-H), 8.29 (d, *J* = 7.6 Hz, 1H, indole C_4_-H), 8.61 (s, 1H, imine H), 11.42 and 11.55 (each s, 1H, NH); IR (cm^-1^): 3331, 1618, 1597, 1302, 837, 752. ESI-MS *m/z*: 278 (M + H^+^). Anal.Calcd for C_17_H_15_N_3_O: C, 73.63; H, 5.45; N, 15.15. Found: C, 73.70; H, 5.47; N, 15.12.

*5-Chloroindole-3-carboxaldehyde 3-chlorobenzoylhydrazone (3b)*

Yield 71%,m.p. 230-233°C^1^HNMR (400 MHz, DMSO-*d*_6_)*δ:*7.22 (d, *J* = 8.4 Hz, *J* = 2.4 Hz, 1H, indole C_6_-H), 7.46 (d, *J* = 8.4 Hz, 1H, indole C_7_-H), 7.56 (t, *J* = 8.0 Hz, 1H, Ar C_5_-H), 7.64 (d, *J* = 8.0 Hz, 1H, Ar C_4_-H), 7.88 (d, *J* = 8.0 Hz, 1H, Ar C_6_-H), 7.92 (s, 1H, indole C_2_-H), 7.96 (s, 1H, Ar C_2_-H), 8.30 (d, *J* = 2.4 Hz, 1H, indole C_4_-H), 8.58 (s, 1H, imine H), 11.66 and 11.77 (each s, 1H, NH); IR (cm^-1^): 3322, 1625, 1602, 1357, 785. ESI-MS *m/z:* 332, 334, 336 (M + H^+^). Anal.Calcd for C_16_H_11_Cl_2_N_3_O: C, 57.85; H, 3.34; N, 12.65. Found: C, 57.70; H, 3.35; N, 12.62.

*5-Chloroindole-3-carboxaldehyde 4-nitrobenzoylhydrazone (3d)*

Yield 66%,m.p. > 270°C.^1^HNMR (400 MHz, DMSO-*d*_6_)*δ*: 7.22 (dd, *J* = 8.4 Hz, *J* = 2.0 Hz, 1H, indole C_6_-H), 7.47 (d, *J* = 8.4 Hz, 1H, indole C_7_-H), 7.95 (s, 1H, indole C_2_-H), 8.15 (d, *J* = 8.4 Hz, 2H, Ar C_3,5_-H), 8.31 (d, *J* = 2.0 Hz, 1H, indole C_4_-H), 8.37 (d, *J* = 8.4 Hz, 2H, Ar C_2,6_-H), 8.61 (s, 1H, imine H), 11.83 (s, 2H, NH); IR (cm^-1^): 3396, 1662, 1518, 1345, 1107. ESI-MS *m/z:* 343, 345 (M + H^+^). Anal.Calcd for C_16_H_11_ClN_4_O_3_: C, 56.07; H, 3.23; N, 16.35. Found: C, 56.13; H, 3.23; N, 16.34.

*5-Chloroindole-3-carboxaldehyde 3-methylbenzoylhydrazone (3e)*

Yield 69%,m.p. 226-228°C.^1^HNMR (400 MHz, DMSO-*d*_6_)*δ*: 2.39 (s, 3H, CH_3_), 7.21 (dd, *J* = 8.8 Hz, *J* = 2.0 Hz, 1H, indole C_6_-H), 7.39 (m, 2H, Ar H), 7.46 (d, *J* = 8.8 Hz, 1H, indole C_7_-H), 7.71 (m, 2H, Ar H), 7.89 (s, 1H, indole C_2_-H), 8.31 (d, *J* = 2.0 Hz, 1H, indole C_4_-H), 8.59 (s, 1H, imine H), 11.52 and 11.75 (each s, 1H, NH); IR (cm^-1^): 3321, 1622, 1601, 1657, 798. ESI-MS *m/z*: 312, 314 (M + H^+^). Anal.Calcd for C_17_H_14_ClN_3_O: C, 65.49; H, 4.53; N, 13.48. Found: C, 65.57; H, 4.54; N, 13.46.

*5-Chloroindole-3-carboxaldehyde 4-methylbenzoylhydrazone (3f)*

Yield 59%,m.p. 244°C (dec.).^1^HNMR (400 MHz, DMSO-*d*_6_)*δ:*2.38 (s, 3H, CH_3_), 7.21 (d, *J* = 8.8 Hz, *J* = 1.6 Hz, 1H, indole C_6_-H), 7.32 (d, *J* = 8.0 Hz, 2H, Ar C_3,5_-H), 7.46 (d, *J* = 8.8 Hz, 1H, indole C_7_-H), 7.82 (d, *J* = 8.0 Hz, 2H, Ar C_2,6_-H), 7.89 (s, 1H, indole C_2_-H), 8.31 (d, *J* = 1.6 Hz, 1H, indole C_4_-H), 8.59 (s, 1H, imine H), 11.49 and 11.74 (each s, 1H, NH); IR (cm^-1^): 3177, 1630, 1610, 1384, 1103, 891. ESI-MS *m/z*: 312, 314 (M + H^+^). Anal.Calcd for C_17_H_14_ClN_3_O: C, 65.49; H, 4.53; N, 13.48. Found: C, 65.55; H, 4.54; N, 13.43.

*5-Chloroindole-3-carboxaldehyde isonicotinoylhydrazone (3g)*

Yield 73%,m.p. > 270°C.^1^HNMR (400 MHz, DMSO-*d*_6_)*δ:*7.22 (dd, *J* = 8.8 Hz, *J* = 2.0 Hz, 1H, indole C_6_-H), 7.47 (d, *J* = 8.8 Hz, 1H, indole C_7_-H), 7.82 (d, *J* = 6.0 Hz, 2H, pyridine C_2,6_-H), 7.94 (s, 1H, indole C_2_-H), 8.30 (d, *J* = 2.0 Hz, 1H, indole C_4_-H), 8.60 (s, 1H, imine H), 8.77 (d, *J* = 6.0 Hz, 2H, pyridine C_3,5_-H), 11.79 (s, 2H, NH); IR (cm^-1^): 1661, 1412, 1127, 889, 782, 749. ESI-MS *m/z*: 299, 301 (M + H^+^). Anal.Calcd for C_15_H_11_ClN_4_O: C, 60.31; H, 3.71; N, 18.76. Found: C, 60.20; H, 3.70; N, 18.81.

*In vitro evaluation of antiplatelet aggregation activity*

The in vitro antiplatelet activity of the derivatives was evaluated using human platelet rich plasma (PRP)on an APACT 4004 aggregometerby Born’s reported turbidimetric method(16). ADP (5 µM), arachidonic acid (1.25 mg/mL) and collagen (5 µM) were used as inducers of platelet aggregation. IC_50_ was defined as the concentration of the test compound that inhibits the platelet aggregation by 50%. The detailed procedure has been described in our previous works(11,12,17).

*Cytotoxicity assay*

The synthesized derivatives were assayed for their toxicity on Fibroblast L929 cell line by MTT [3-(4,5-dimethylthiazol-2-yl-2,5-tetrazolium bromide)] method. The compounds were initially screened at 100 µM concentration and IC_50 _was calculated for those showing more than 50% toxicity. The detailed procedure has been described in our previous work(18).

*Platelet lactate dehydrogenase (LDH) assay*

The assay was performed according to the previously reported procedures(19). Briefly, PRP samples (200 µL) were incubated with test solutions (1 µL, final concentration of 100 µM) for 30 min. The samples were then centrifugatedat 11000 rpm for 5 min and the supernatant was separated. The activity of lactate dehydrogenase was evaluated using the supernatant with an LDH assay kit (Pishtazteb, Iran) following the instructions provided by the manufacturer. DMSO and 1% Triton X-100 were used as vehicle and positive control respectively. The data was expressed as the proportion of released LDH following exposure to test compounds to the total LDH content measured by the addition of 1% Triton X-100.

## Results and Discussion

*Chemistry*

**Scheme 1:**
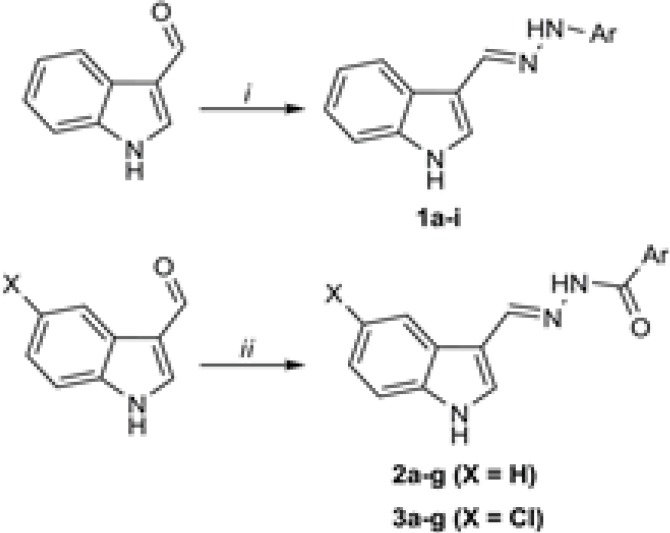
Synthetic route to the hydrazone derivatives; reagents and conditions: (*i*) arylhydrazines, ethanol, glacial acetic acid, reflux; (*ii*) aroylhydrazides, ethanol, glacial acetic acid, reflux.

As disclosed in scheme 1, the final hydrazones were prepared by a single step procedure. To this end, (5-chloro)indole-3-carboxaldehyde was reacted to different arylhydrazines (compounds 1a-i) or aroylhydrazides (compounds 2a-g and 3a-g). The reactions were performed in ethanol using catalytic amounts of glacial acetic acid. Notably, the aroylhydrazides were purchased from the commercial suppliers or otherwise synthesized from their starting benzoic acids following the previously reported methods(20). Following the typical procedure (as described in the experimental section) the final hydrazone derivatives were prepared in good yields with excellent analytical purity as confirmed by thin layer chromatography and CHN elemental analysis. 

The spectral data were in complete agreement with the structure of the derivatives. In the ^1^H-NMR spectra, the hydrogens of the indole and 5-chloroindole nucleus resonated by a characteristic pattern. In compounds 1a-i and 2a-gindolehydrogensappeared as a set of multiplet in 7.10-7.25 ppm (assigned to indole H-5 and H-6), two sets of doublets at ~ 7.46 ppm and ~ 8.30 ppm (assigned to indole H-7 and H-4) and a singlet at 7.8-8.0 ppm (assigned to indole H-2). In compounds 3a-g, two singlets at7.8-8.0 ppm and ~ 8.30 ppm and two sets of doublets at ~ 7.21 ppm and ~ 7.46 ppm were the characteristic picks for 5-chloroindole nucleus. The aromatic hydrogens on the aryl(aroyl)hydrazone moiety were readily distinguishable in the ^1^H-NMR spectra of the derivatives according to their multiplicity patterns. Analysis of the molecular mass of the derivatives was performed by ESI-MS and molecular ions of the compounds were observed as adducts of hydrogen and/or sodium.

*Biological activity*

The antiplatelet activity of the derivatives has been disclosed in table 1. All the compounds were evaluated for their in vitro antiplatelet activity using arachidonic acid (AA), ADP and collagen as inducers of platelet aggregation. At the first view, it could be noted that the arylhydrazone derivatives 1a-i showed considerable activity against AA- and collagen-induced platelet aggregation and showed no activity when ADP was used as aggregation inducer. However, aroylhydrazones 2a-g and 3a-gwere completely inactive. It should be considered that Hydrazones contain two connected nitrogen atoms of different nature and a C-N double bond that is conjugated with a lone electron pair of the terminal nitrogen atom. These structural fragments are mainly responsible for the physical and chemical properties of hydrazones. Both nitrogen atoms of the hydrazine group are nucleophilic, although the amino type nitrogen is most reactive. Despite the high structural similarity between hydrazones and carbohydrazones, the electronic characteristics of these compounds are quite different: the presence of carbonyl, conjugated with the lone pair of electrons will change the delocalization pattern of electrons on carbohydrazone derivatives.

**Table 1:** Antiplatelet activity and cytotoxicity of the derivatives

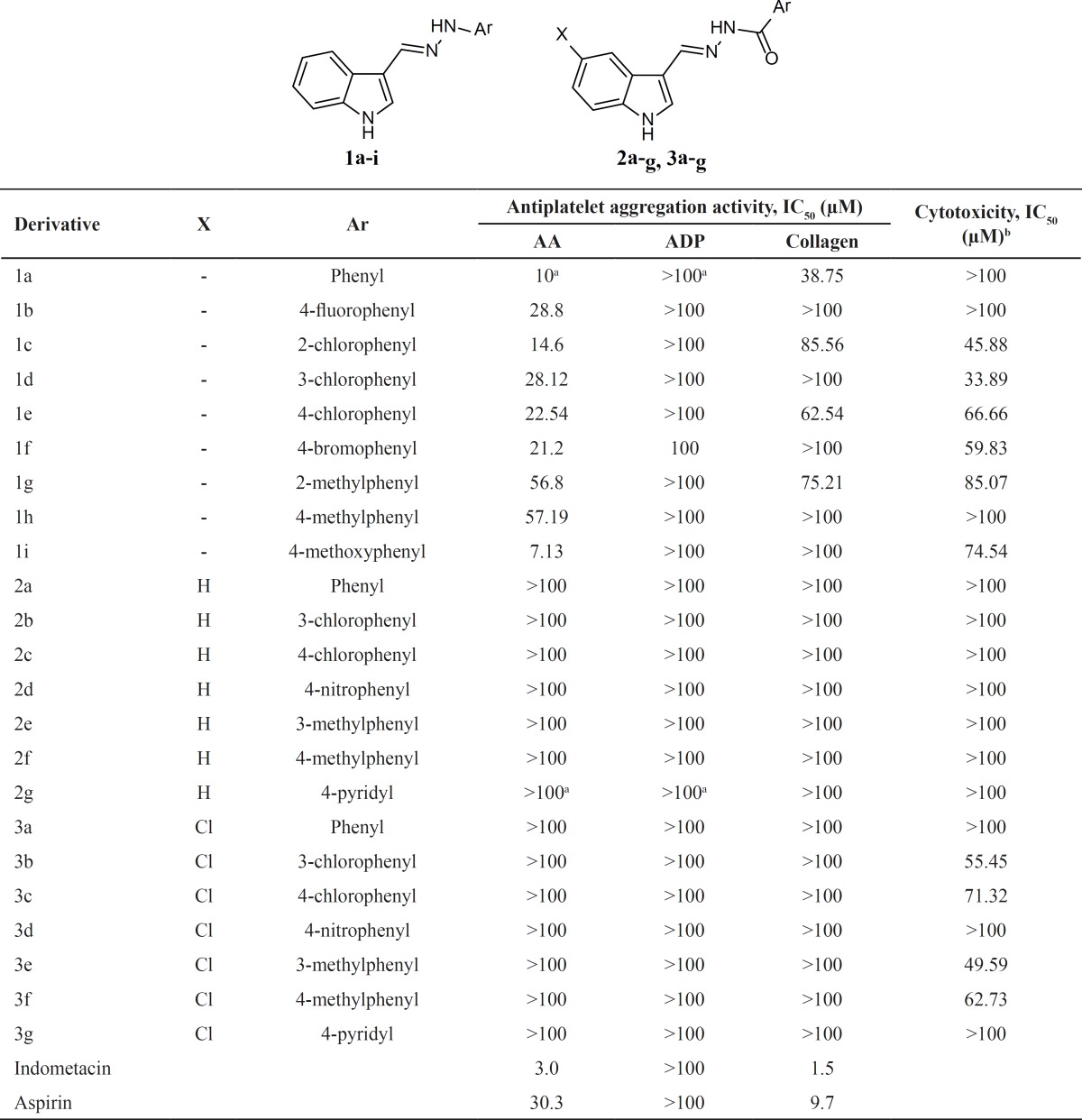

a Obtained in our previous study (11)

b Assayed against fibroblast L929 cell line after 24 h of exposure.

In carbohydrazones, the amide-type resonance between the nitrogen and carbonyl alters the electron density so that the complexationbehavior of these compounds differ substantially from that of hydrazones. Therefore the striking difference between the biological activities of hydrazine derivatives and their carbohydrazone congeners could be on account of the different electronic interaction of these two group of compounds with their receptors. It is also worth mentioning that the companionship of carbonyl moiety in carbohydrazone derivatives will also change the shape and size of the molecules. 

In the arylhydrazone series, halogen and methoxy substituted derivatives exhibited acceptable activity against AA-induced platelet aggregation. Compound 1i was the most potent derivative in the current study with IC_50_ value of 7.13 µM which was lower that its parent compound (1a). The activity of compound 1c (R = 2-chlorophenyl) and 1f (4-bromophenyl) was still acceptable.Bioevaluation of the synthesized derivatives against collagen-induced platelet aggregation revealed that some arylhydrazones including 1a, 1e and 1g showed moderate activity. Among them, compound 1a was the most active one with IC_50_ value of 38.75 µM. 

The global physicochemical parameters for the active compounds 1a-i including ClogP, refractivity, polarizability, molecular volume and surface area were calculated and the results were presented in table 2. Analysis of the activity data and calculated physicochemical parametersshows a weak correlation between IC_50_ values for AA-induced platelet aggregationand lipophilicity(CLogP). According to table 2, the most active compounds including 1a and 1i have the lowest calculated LogP values in the arylhydrazone series; and as a general trend, the increase of lipophilicity is accompanied by reduction of bioactivity. As mentioned above, this implies that lipophilicity may play a role to some extents in the antiplatelet activity of compounds 1a-i. 

**Table 2:** Global physicochemical parameters calculated for compounds 1a-i.

**Derivative**	**CLog** ***P***	**R**	**P**	**V**	**SA**	
					**Approx**	**Grid**
1a	4.13	74.01	29.04	728.65	350.18	450.79
1b	4.27	74.22	28.94	732.23	356.62	452.41
1c	4.65	78.81	30.96	768.53	376.46	474.03
1d	4.65	78.81	30.96	781.96	392.35	486.85
1e	4.65	78.81	30.96	772.99	385.48	473.83
1f	4.92	81.63	31.66	792.01	394.56	485.00
1g	4.60	79.05	30.87	782.24	382.59	485.04
1h	4.60	79.05	30.87	781.82	393.27	478.63
1i	3.88	80.47	31.51	806.46	408.80	495.65

Cytotoxicity evaluation of a drug candidate is indeed crucial to its fate in lead identification and the following phases of drug discovery process. Therefore, we examined the cytotoxicity of the hydrazone derivatives on fibroblast L929 cells as a normal cell line and also on platelet concentrates. As shown in table 1, compounds 1a, 1b and 1h showed antiplatelet activity at concentrations which were not toxic to fibroblast cells. In addition, compound 1i as the most potent derivative, showed an acceptable selectivity. Besides the fibroblast cells, the safety of the active arylhydrazone derivatives 1a-i was evaluated on platelet cells. It is known that platelet membrane damage leads to the leakage of lactate dehydrogenase (LDH) from the platelet cytosol to the surrounding medium and the amount of released LDH is proportional to the damaged platelets (19). Therefore, platelet LDH assay is widely used to evaluate the effect of drugs and xenobiotics on platelets(19,22-24). Our experiments showed that incubation of platelet with test compounds did not lead to a significant LDH release as compared to vehicle. This shows the safety of antiplatelet arylhydrazonesto the platelet cells.

## Conclusion

The present work describes the synthesis and antiplatelet aggregation activity of a series of aryl(aroyl)hydrazone derivatives which were designed by considering indole-3-carboxaldehyde 1a as lead compound. The activity data showed the complete loss of bioactivity when arylhydrazone fragment was replaced by aroylhydrazone. Similar to compound 1a, the other arylhydrazones showed inhibitory activity against AA- and collagen-induced platelet aggregation. Among them, compound 1i proved to be the most potent antiplatelet derivative with an IC_50_ value comparable to that of indomethacin as a standard inhibitor of AA metabolism. In addition, toxicity assessment of the hydrazone derivatives revealed that compounds 1a-i possess an acceptable selectivity towards the antiplatelet activity.
